# Results and lessons learnt from the WISTERIA phase I trial combining AZD1775 with cisplatin pre- or post-operatively in head and neck cancer

**DOI:** 10.1038/s44276-023-00026-6

**Published:** 2024-01-29

**Authors:** Anthony Kong, Amanda J. Kirkham, Joshua S. Savage, Rhys Mant, Siân Lax, James Good, Martin D. Forster, Joseph J. Sacco, Stephano Schipani, Kevin J. Harrington, Christina Yap, Hisham Mehanna

**Affiliations:** 1 King’s College London, London, UK; 2 Cancer Research UK Clinical Trials Unit, Institute of Cancer and Genomics Sciences, University of Birmingham, Birmingham, UK; 3 University Hospitals Birmingham NHS Foundation Trust, Birmingham, UK; 4 UCL Cancer Institute / University College London Hospitals NHS Foundation Trust, London, UK; 5 The Clatterbridge Cancer Centre, Wirral/University of Liverpool, Liverpool, UK; 6 Beatson West of Scotland Cancer Centre, Institute of Cancer Sciences, University of Glasgow, Glasgow, UK; 7 The Institute of Cancer Research, London, UK; 8 Clinical Trials and Statistics Unit, The Institute of Cancer Research, London, UK; 9 InHANSE, Institute of Cancer and Genomic Sciences, University of Birmingham, Birmingham, UK

## Abstract

**Background:**

Pre-clinical studies suggest AZD1775, a WEE1 kinase inhibitor, potentiates the activity of various chemotherapeutic agents.

**Methods:**

WISTERIA was a prospective, parallel two-group, open-label, dose-finding, phase I clinical trial. Eligible patients had histologically confirmed oral, laryngeal, or hypopharyngeal squamous cell carcinoma, ECOG performance status 0/1, and aged ≥18-to-≤70 years. Primary outcomes were adverse events and defining recommended dose and schedule of AZD1775 in combination with cisplatin in pre-operative (Group A), or with cisplatin/radiotherapy in post-operative (Group B) patients. Dose determination was guided by a modified time-to-event continual reassessment method (mTITE-CRM).

**Results:**

Between 30-Oct-2017 and 15-Jul-2019, nine patients were registered: Three into Group A and six into Group B. WISTERIA was closed early due to poor recruitment. Five dose-limiting toxicities (DLTs) were reported in four Group B patients. Seven serious adverse events were reported in four patients: One in Group A, and three in Group B. Three were related to treatment. No treatment-related deaths were reported.

**Conclusions:**

WISTERIA did not complete its primary objectives due to poor recruitment and toxicities reported in Group B. However, use of the novel mTITE-CRM improved flexibility in reducing accrual suspension periods and should be considered for future trials in complex patient populations.

**Clinical Trial Registration:**

ISRCTN76291951

## Introduction

Head and neck squamous cell carcinoma (HNSCC) is the sixth most common cancer worldwide, with over 12,000 reported cases of locally advanced laryngeal, oral and hypopharyngeal cancer each year in the UK between 2016 and 2018 [1]. Combined modality treatment with surgery, radiotherapy and/or chemotherapy is the standard-of-care, with post-operative radiotherapy (PORT) recommended for patients with locally advanced disease and those who have poor prognostic histological features after surgical resection e.g., perineural/vascular invasion, or multiple involved lymph nodes. Platinum-based post-operative chemo-radiation (POCRT) is specifically recommended for those with involved margins or those with extra-capsular spread (ECS) of disease in involved lymph nodes [2].

Despite this intensive treatment, three-year overall survival is sub-optimal at 60–70%. Loco-regional relapse is particularly difficult to salvage, and local control is closely correlated with overall survival as are higher quality of life (QoL) scores. Therefore, there remains an urgent need to develop novel approaches that achieve improved loco-regional disease control for this patient group, which may translate into improved overall survival and an enhancement in patient-related outcome measures.

POCRT exploits the cellular DNA damage response (DDR) in malignant and normal tissues to eradicate microscopic residual disease. Cell cycle checkpoints are an integral and druggable component of the DDR, allowing the cell to pause and repair the DNA. Mutations in TP53, a key regulator of the G1/S checkpoint are seen in 60-70% of HNSCC cases [3], and are sufficient to impair the function of this checkpoint, and thereby create a critical reliance on the later G2/M checkpoint. Pharmacological abrogation of the G2/M checkpoint has been shown to differentially sensitise normal and tumour cells to the effects of DNA-damaging agents such as cisplatin and ionising radiation (IR) [4].

WEE1 kinase is a key regulator of the G2/M checkpoint and a promising therapeutic target. It is a serine-threonine kinase involved in phosphorylation and inactivation of cyclin-dependent kinase (CDK)1 and CDK2 and has been implicated in maintaining genomic stability through stabilisation of replication forks [5]. WEE1 upregulation is seen in a variety of human cancers and is inversely associated with prognosis in some models [6, 7]. Kinomiescreens in HNSCC have identified WEE1 expression as a strong determinant of cell survival [8, 9].

AZD1775 is a potent, selective small molecule inhibitor of WEE1. Several pre-clinical studies have suggested that AZD1775 potentiates the activity of various chemotherapeutic agents [10–15], including cisplatin-induced G2/M arrest in HNSCC TP53 mutant cell lines [16]. Furthermore, data suggest that p53 mutation is a predictive biomarker for response to WEE1 inhibition by AZD1775 [17].

At the time of this trial’s inception, AZD1775 had shown single-agent activity in patients carrying BRCA mutations [18] and was being tested in combination with radiotherapy in childhood pontine glioma (NCT01922076), with temozolomide and radiotherapy in glioblastoma (NCT01849146), and with cisplatin and radiotherapy in cervical cancer (NCT01958658).

Swift evaluation of novel radiotherapy-drug combinations in complex clinical settings has been limited by the periodic suspension of accrual whilst patients complete follow-up to assess the occurrence of dose-limiting toxicities (DLTs) [19, 20]. The risk of potential delayed-onset toxicities (a particular challenge for phase I trials with radiotherapy combinations), makes conventional rule-based designs result in infeasible lengthy trial durations within the funding requirements (in terms of both time and cost) or, indeed, the patent-life of novel agents. Based on clinical, biological, and statistical considerations, WISTERIA (ISRCTN76291951/NCT03028766) was designed as a two-part trial to incorporate AZD1775 treatment in those HNSCC patients of the oral cavity, larynx and hypopharynx who were planned to undergo surgical resection in both the pre- and post-surgical settings conducted simultaneously. The aims were to determine the safety profile through the use of an efficient Bayesian Time-to-event Continual Reassessment Method (TITE-CRM) [21] to identify the (a) maximum tolerated dose (MTD) of AZD1775 in combination with a single dose of cisplatin pre-operatively as a window-of-opportunity trial (Group A); and (b) MTD of AZD1775 in combination with cisplatin/radiotherapy post-operatively (Group B).

## Methods

### Trial design

WISTERIA was a parallel two-group, open-label, dose-finding, phase I clinical trial recruiting patients from six hospitals in the UK [22].

### TITE-CRM model for MTD assessment

As previously described [22], the modified Bayesian TITE-CRM design used an empiric dose-toxicity model requiring a maximum of 21 patients per group and encompassed up to four dose levels of AZD1775. Predefined dose-limiting toxicities (DLTs) were specified by the clinical investigators of WISTERIA and have previously been described in full [22] and summarised in Supplementary Appendix A.

TITE-CRM models were tested for both Group A and Group B separately. The corresponding operating characteristics and dose transition pathways were obtained through simulation studies and are provided in Supplementary Appendix B.

Two sensitivity analyses were performed to determine if the amount of treatment received would influence the TITE-CRM model decision: A 50:50 weighted dose and time model, and a 40:60 weighted model. Both sensitivity analyses derived similar posterior probability values (to three decimal places) as those obtained from the non-treatment-adjusted TITE-CRM model results. Details of the algorithm adjustment can be found in Supplementary Appendix C. On comparing the outputs from both treatment-adjusted TITE-CRM models and the non-treatment-adjusted TITE-CRM model, it was observed that accounting for the amount of trial treatment (AZD1775) received by each patient had very little effect on the TITE-CRM model outcome and recommendation for the next dose was similar for all TITE-CRM models applied. These results were presented to the Trial Safety Committee (TSC).

### Dose decision-making committee

The independent TSC, composed of external clinicians and one statistician, reviewed interim data once each cohort of patients had been recruited and assessed DLTs within the defined assessment timeframe. Additional meetings were convened if late onset DLTs were observed. The TSC was responsible for decisions relating to changing the recommended treatment dose as indicated by the modified TITE-CRM model.

### Patient eligibility

Eligible patients had histologically confirmed oral, laryngeal or hypopharyngeal squamous cell carcinoma, Eastern Cooperative Oncology Group (ECOG) performance status 0/1, and were aged ≥18 to ≤70 years. Group A patients required accessible tumours for re-biopsy under local anaesthetic or via ultrasound-guided biopsy. Group B patients had high-risk histopathological features requiring treatment with post-operative chemoradiotherapy after surgical resection. Full criteria were previously published [22].

Patient registration by the treating clinician was by telephone to the Cancer Research UK Clinical Trials Unit (CRCTU).

### Interventions and procedures

As previously detailed in Fig. 1 of Kong et al. [22], Group A (pre-operative) patients received the cohort-specified dose of oral AZD1775 bd for three days, commencing on both days one and eight, with 40 mg/m^2^ intravenous (IV) cisplatin delivered on day eight. Group B (post-operative) patients received the cohort-specified dose of oral AZD1775 bd for three days, commencing on days two, nine, 23 and 30, with 40 mg/m^2^ IV cisplatin delivered on days two, nine, 16, 23 and 30, where days were timed from the start of radiotherapy delivery. Radiotherapy (54–65 Gγ in 30 fractions) was given concurrently with chemotherapy over six weeks commencing within three months of surgery.

**Fig. 1:**
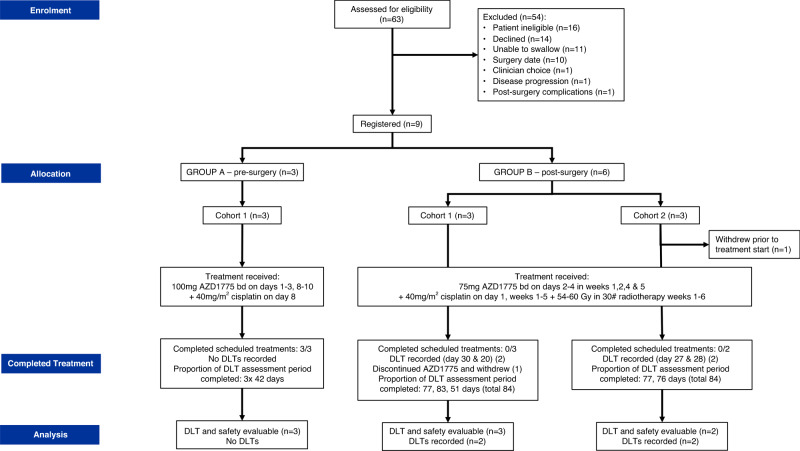
WISTERIA trial profile. The trial profiles of the two groups and three cohorts analysed in the WISTERIA trial. DLT dose-limiting toxicity.

Patients in Group A were followed up four and 12 weeks after treatment ended, with those in Group B weekly for four weeks following the end of treatment, at 12 weeks, and six and 12 months.

### Outcomes

Co-primary outcomes were to determine the recommended dose and schedules for further testing and safety profile of AZD1775 in combination with cisplatin in the pre-operative (window of opportunity) setting (Group A), and with cisplatin/radiotherapy in the post-operative setting (Group B) as determined by a modified TITE-CRM [21, 23–25]. The safety profile of all patients was determined as per Common Terminology Criteria for Adverse Events (CTCAE) v4.0 [26]. The secondary outcome was to obtain preliminary data about disease-free survival from the start of treatment to the date of disease recurrence, patient death or end of follow-up.

Tertiary outcomes included evaluation of the pharmacodynamic effects of AZD1775, and to identify and correlate potentially predictive biomarkers with pharmacodynamic markers of DNA damage as previously published [22]. Finally, overall QoL and head and neck-specific QoL was assessed for patients in Group B using EORTC C30 [27], EORTC QLQ-H&N35 [28], and M. D. Anderson Dysphagia Inventory (MDADI) [29]. Patients completed questionnaires independently prior to commencement of radiotherapy, at the end of treatment assessment, and at the 12-week, six- and 12-month follow-up visits. Due to the early stopping of the trial, analyses of these data were limited.

### Statistical analysis

MTD was defined as the dose level with an estimated DLT rate closest to the target DLT rate of 25% and 30% for Group A and Group B, respectively, and determined using the respective modified TITE-CRM models. Parameters obtained from the models are presented and graphically displayed. Additional sensitivity analyses were performed for Group B results using treatment-adjusted TITE-CRM models to verify if results for participants not receiving the full treatment would influence the decision obtained from the TITE-CRM models.

Median disease-free survival and corresponding 95% confidence intervals were planned using Kaplan and Meier.

Analyses were performed using Stata v17.0 and R v4.1.0.

## Results

WISTERIA was closed early due to poor recruitment, and high toxicity rates in combination with CRT in Group B. Between 30-Oct-2017 and 15-Jul-2019, nine patients were registered: three in Group A and six in Group B (Fig. 1). Two patients from Group B withdrew from the trial; one 20 days post-registration having received the first two weeks of AZD1775 (75 mg), and a second five days post-registration prior to receiving any trial treatment. No patient deaths were reported.

Patient characteristics are described in Table 1. The median age for patients in the trial was 59 years (range 49 to 64) with 5/9 male and 7/9 having ECOG performance status 0.

**Table 1:** Patient baseline characteristics.

Characteristic	Treatment Group		Overall
	Group A - Pre-surgery	Group B – Post-surgery	
	*N* = 3	*N* = 6	*N* = 9
Sex			
Male	1	4	5
Female	2	2	4
Age			
Mean (s.d.)	52.3 (4.2)	61.0 (2.3)	58.1 (5.1)
Median	51.0	61.0	59.0
IQR	49.0, 57.0	59.0, 63.0	57.0, 61.0
Range	49.0, 57.0	58.0, 64.0	49.0, 64.0
ECOG			
0	3	4	7
1	0	2	2
Tumour Types			
Oral cavity	3	4	7
Hypopharynx larynx	0	1	1
Larynx	0	1	1
Side of Tumour			
Left	2	4	6
Right	1	2	3
Tumour Differentiation			
Moderate	3	5	8
Poor	0	1	1
Histology Type			
Squamous cell carcinoma	3	6	9
Imaging Stage			
T			
T2	1	0	1
T4a	1	0	1
Not known	1	0	1
Not applicable	0	6	6
Total	3	6	9
N			
N0	1	0	1
N2b	1	0	1
N2c	1	0	1
Not applicable	0	6	6
M			
M0	1	0	1
Mx	2	0	2
Not applicable	0	6	6

A table of the patient baseline characteristics within the WISTERIA trial.

*IQR* interquartile range, *s.d.* standard deviation.

**Fig. 2:**
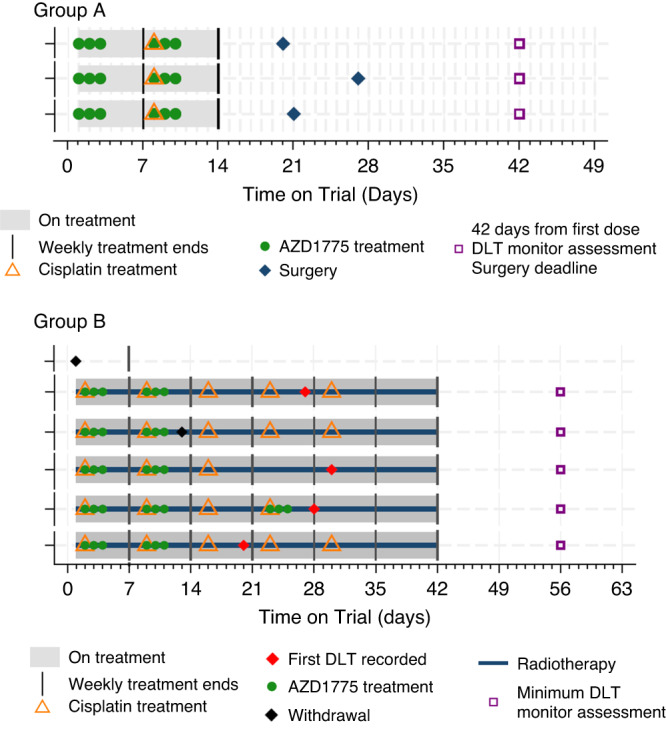
Treatment pathways of registered patients. Swimmer plots detailing the treatment pathways of patients registered to the WISTERIA trial. See Supplementary Appendix D for the patients’ full pathways including follow-up. All patients in Group A received 100 mg AZD1775 bd for three days during weeks one and two. Four patients in Group B received 75 mg AZD1775 bd for three days during weeks one and two. One patient received 75 mg AZD1775 bd for three days during weeks one, two, and four. One patient withdrew from the trial prior to receiving any treatment.

Details of each patient’s on-trial journey are summarised in Fig. 2 and Supplementary Appendix D.

Three patients in Group A received 100 mg AZD1775 bd for 3 days as per protocol dose level 0 although one patient recorded a delay with their ninth dose and another patient recorded a delay in receiving their tenth dose; both received antiemetics during weeks one and two (Fig. 2). Cisplatin was given as scheduled, with no patient treated with carboplatin, and all patients underwent scheduled surgery within the pre-specified 42 days from start of treatment. All participants completed the full DLT monitoring period and no DLTs were reported.

Supplementary Appendix E describes using the modified TITE-CRM, which was updated following the incorporation of this initial three-patient Group A Cohort, the dose level with the closest posterior probability estimate to the target DLT rate of 25% was predicted to be 150 mg (dose level 2). As the modified TITE-CRM did not permit skipping of untried dose levels, the next recommended dose for Group A Cohort 2 was 125 mg (dose level 1), but this was not explored as the trial was stopped early.

The first three patients registered into Group B received 75 mg bd AZD1775. Following the TSC review, a further three patients were registered into Cohort 2 at the same dose (75 mg bd AZD1775) (see Supplementary Appendix E). One patient withdrew from the trial before receiving any treatment (Fig. 2). Of the five evaluable Group B patients, four experienced five DLTs (Table 2). All five patients discontinued treatment (Tables 2 and 3).

**Table 2:** Summary of treatment and dose-limiting toxicities.

Days on Trial(days)	Cohort	Days on Treatment	Weeks Given AZD1775	Discontinued AZD1775	Weeks Given Cisplatin	Discontinued Cisplatin	Radiotherapy Completed	CTCAE Toxicity	DLT	Inability to Swallow
Group A										
100	1	10	1, 2	No	–	No	–	–	No	–
99	1	10	1, 2	No	–	No	–	–	No	–
98	1	10	1, 2	No	–	No	–	–	No	–
Group B										
384	1	20	1, 2	Yes	1 to 5	No	Yes	Dysphagia	Yes	Yes
370	2	43	1, 2, 4	Yes	1 to 4	Yes	Yes	Febrile neutropenia	Yes^^^	No
364	1	36	1, 2	Yes	1 to 3	No	Yes	Neutrophil count decreased Mucositis	Yes Yes	No
363*	1	20	1, 2	Yes	1 to 5	No	Yes	-	No	–
350	2	32	1, 2	Yes	1 to 5	No	Yes	Mucositis	Yes	Yes

A summary of treatment received and dose limiting toxicities (DLTs) experienced by patients during the WISTERIA trial.

*Patient chose to withdraw from the trial due to the burden of ongoing treatment

^^^This DLT was also reported as a serious adverse event (see Table 3).

One patient in Group B withdrew from the trial before receiving AZD1775 so is not included in this table.

**Table 3:** Serious adverse event details.

Days on Trial	Category	Event	Duration (Days)	Outcome
Group A				
100	Unrelated SAE	Mucositis	5	Resolved–with sequelae
100	Unrelated SAE	Mucositis	3	Resolved–with sequelae
Group B				
384	Non-fatal/life-threatening SUSAR	Dysphagia	18	Resolved–no sequelae
384	SAR	Diarrhoea	3	Resolved–no sequelae
384	SAR	Nausea	2	Resolved–with sequelae
370*	SAR	Febrile neutropenia	27	Resolved–no sequelae
350	Unrelated SAE	Skin Infection	2	Resolved–no sequelae

A list of all serious adverse events that occurred during the WISTERIA trial.

*SAE* serious adverse event, *SAR* serious adverse reaction (i.e., drug-related), *SUSAR* suspected unexpected serious adverse reaction.

*This SAE was also reported as a dose limiting toxicity (see Table 2).

Analysis of all five evaluable Group B patients was performed using the modified TITE-CRM. The dose level with the closest posterior probability estimate to the target DLT rate of 0.30 (30%) was 50 mg (dose level -1) (see Supplementary Appendix E). Therefore, the TITE-CRM model recommended reducing the dose to 50 mg AZD1775 for the next cohort. Considering the slow recruitment rate and toxicity demonstrated at relatively low levels of the drug in Group B, a decision was taken by the TMG to end recruitment into the trial and approved by the TSC.

In total, there were seven SAEs reported in four patients during the trial: One patient in Group A and three in Group B (Table 3). In total, there were 176 AEs; 44 in Group A, and 132 in Group B (Table 4).

**Table 4:** Summary of adverse events.

	CTCAE Grade				
Adverse Event Category (*N* (%))	Grade 1	Grade 2	Grade 3	Grade 4	Overall
Group A					
Blood and lymphatic system disorders	0 (0.0)	0 (0.0)	3 (30.0)	0 (0.0)	3 (6.8)
Cardiac disorders	1 (7.1)	1 (5.0)	0 (0.0)	0 (0.0)	2 (4.5)
Gastrointestinal disorders	6 (42.9)	11 (55.0)	1 (10.0)	0 (0.0)	18 (40.9)
General disorders and administration site conditions	1 (7.1)	2 (10.0)	0 (0.0)	0 (0.0)	3 (6.8)
Infections and infestations	0 (0.0)	2 (10.0)	0 (0.0)	0 (0.0)	2 (4.5)
Injury, poisoning and procedural complications	1 (7.1)	2 (10.0)	0 (0.0)	0 (0.0)	3 (6.8)
Investigations	1 (7.1)	0 (0.0)	2 (20.0)	0 (0.0)	3 (6.8)
Metabolism and nutrition disorders	2 (14.3)	0 (0.0)	4 (40.0)	0 (0.0)	6 (13.6)
Nervous system disorders	1 (7.1)	0 (0.0)	0 (0.0)	0 (0.0)	1 (2.3)
Psychiatric disorders	1 (7.1)	1 (5.0)	0 (0.0)	0 (0.0)	2 (4.5)
Respiratory, thoracic and mediastinal disorders	0 (0.0)	1 (5.0)	0 (0.0)	0 (0.0)	1 (2.3)
Total	14	20	10	0	44
Group B					
Blood and lymphatic system disorders	3 (4.8)	5 (10.6)	2 (9.5)	1 (100.0)	11 (8.3)
Ear and labyrinth disorders	1 (1.6)	1 (2.1)	0 (0.0)	0 (0.0)	2 (1.5)
Gastrointestinal disorders	27 (42.9)	20 (42.6)	5 (23.8)	0 (0.0)	52 (39.4)
General disorders and administration site conditions	6 (9.5)	5 (10.6)	1 (4.8)	0 (0.0)	12 (9.1)
Infections and infestations	0 (0.0)	1 (2.1)	0 (0.0)	0 (0.0)	1 (0.8)
Injury, poisoning and procedural complications	3 (4.8)	2 (4.3)	1 (4.8)	0 (0.0)	6 (4.5)
Investigations	1 (1.6)	2 (4.3)	4 (19.0)	0 (0.0)	7 (5.3)
Metabolism and nutrition disorders	10 (15.9)	4 (8.5)	6 (28.6)	0 (0.0)	20 (15.2)
Musculoskeletal and connective tissue disorders	1 (1.6)	0 (0.0)	0 (0.0)	0 (0.0)	1 (0.8)
Nervous system disorders	4 (6.3)	4 (8.5)	0 (0.0)	0 (0.0)	8 (6.1)
Psychiatric disorders	0 (0.0)	1 (2.1)	0 (0.0)	0 (0.0)	1 (0.8)
Respiratory, thoracic and mediastinal disorders	1 (1.6)	1 (2.1)	0 (0.0)	0 (0.0)	2 (1.5)
Skin and subcutaneous tissue disorders	5 (7.9)	1 (2.1)	2 (9.5)	0 (0.0)	8 (6.1)
Surgical and medical procedures	1 (1.6)	0 (0.0)	0 (0.0)	0 (0.0)	1 (0.8)
Total	63	47	21	1	132

A summary of all those adverse events as defined by Common Terminology Criteria for Adverse Events (CTCAE) v4.0 [26] that occurred during the WISTERIA trial.

At the time of data lock (13-Dec-2022), all three patients recruited into Group A were alive with no signs of disease reported, and all five evaluable patients in Group B were alive, with only one reporting local disease recurrence at the primary site before their 12-month follow-up visit. Therefore, median disease-free survival could not be calculated.

Pharmacokinetic analyses demonstrated that the mean change in AZD1775 concentration comparing pre- and post-administration on day 3 for patients in Group A was 113.3% (range 14.2-187.6), and on day 10 pre- and post-AZD1755 administration the mean change was 116.0% (range 23.6–191.3); for patients in Group B, the mean change in AZD1775 concentration comparing pre- and post-AZD1775 administration on day 5 was 110.5% (range 44.0–158.2) (Supplementary Appendix F). Due to the early stopping of the trial, the feasibility of assessing potentially predictive biomarkers was not possible.

Quality of life data were collected for patients in Group B but due to the small number of patients recruited few conclusions can be drawn. As exemplified in the EORTC QLQ C30 Global Health Score, QoL scores reduced during treatment (week one to end of treatment mean score change = –26.3%) and at 12-week follow-up (end of treatment to 12-week follow-up mean score = –6.2%) before slowly increasing (12-week follow-up to six-month mean score change = 29.2%; six-month follow-up to 12-month mean score change = 5.1%) to levels similar to those at pre-treatment; week one mean score = 73.0 (95%CI: 58.56–87.40) compared to 12-month follow-up mean score = 64.6 (95%CI: 48.43–80.77) (Supplementary Appendix G).

## Discussion

In this trial, we conducted a phase Ib trial to assess whether the WEE1 inhibitor AZD1775 could be safely combined with cisplatin chemotherapy pre-operatively (Group A) and with adjuvant concurrent chemoradiation post-operatively (Group B) without excessive acute and late toxicities in HNSCC patients undergoing curative surgery.

In Group A, we originally intended to recruit up to 21 patients in four dose levels but only one dose cohort (100 mg bd, dose level 0) with three patients was recruited before the closure of the trial due to slow recruitment. The TITE-CRM recommended recruitment to the next higher dose level of 125 mg. However, due to the closure of the trial, this could not be undertaken and so the recommended dose and schedule of AZD1775 in combination with cisplatin, could not be determined.

In Group B, we assessed the safety of combining AZD1775 with standard adjuvant chemoradiation in resectable HNSCC patients with high-risk histopathological features including positive margins and/or ECS with a view to improve the outcome for this group of patients by enhancing the effect of chemoradiation. A total of six patients (out of the originally intended 21 patients) were recruited into dose level 0: 75 mg AZD1775 bd for three days, commencing on days two, nine, 23 and 30, with 40 mg/m^2^ IV cisplatin delivered on days two, nine, 16, 23, and 30 with post-operative radiotherapy 54–65 Gy in 30 fractions given over six weeks. There were five DLTs occurring in four of the five evaluable patients (one patient experienced two DLTs). This indicated the potentiation of acute toxicities of adjuvant chemoradiation in combination with AZD1775 even at a low dose, resulting in patients’ inability to complete the intended course of AZD1775 with chemoradiation. The TITE-CRM model recommended reducing the AZD1775 dose to dose level –1 (50 mg AZD1775) for the next cohort, had the trial continued.

In a previous phase I study, the MTD monotherapy dose of AZD1775 in patients with refractory solid tumours was determined to be 225 mg bd for 2.5 days in weeks one and two of a three-week cycle (a total dose of 2250 mg every 3 weeks) [18]. In a second phase Ib study, the MTD dose for AZD1775 was determined to be 200 mg bd for 2.5 days every 21 days (a total dose of 1000 mg every three weeks) with cisplatin 75 mg/m^2^ in patients with advanced solid tumours [30]. Therefore, had we continued the WISTERIA trial, the modified TITE-CRM predicted the MTD dose to be AZD1775 150 mg bd (dose level 2) for three days, on day one as monotherapy (total 900 mg on week one) and day eight in combination with 40 mg/m^2^ cisplatin (total dose 900 mg on week 2) in Group A.

A future study could explore the combination of AZD1775 with cisplatin or with cisplatin and docetaxel as a neoadjuvant regimen, and then be compared with standard induction chemotherapy (cisplatin, docetaxel and 5FU or cisplatin and docetaxel) to assess the anti-tumour efficacy as well as toxicities between the regimens. A previous phase I study demonstrated that AZD1775 bd over 2.5 days on week one given in combination with weekly cisplatin (25 mg/m^2^) and docetaxel (35 mg/m^2^) for three additional weeks as neoadjuvant treatment was suitable for patients with stage III/IVB HNSCC planned for definitive chemoradiation [31]. The MTD for AZD1775 was determined to be 150 mg orally bd for 2.5 days with promising anti-tumour efficacy of the combination with an ORR of 50% and SD of 40% [31].

In HNSCC patients, AZD1775 was previously combined with definitive chemoradiotherapy for patients with intermediate- and high-risk, locally advanced HNSCC in a phase Ib study and the RP2D of AZD1775 was 100 mg (bd on days one to three of weeks one, two, four, five, seven, and eight), in combination with 70 Gy of radiotherapy and concurrent cisplatin 30 mg/m^2^ [32]. Three patients (25% out of 12 enrolled patients) experienced a DLT, including grade 4 thromboembolism and febrile neutropenia [32]. This study was similar to WISTERIA but in patients undergoing definitive chemoradiotherapy rather than post-operative chemoradiotherapy. The use of a lower weekly cisplatin dose of 30 mg/m^2^, compared to the standard weekly dose of 40 mg/m^2^, with the addition of AZD1775 still resulted in a DLT rate of 25%. This is lower than the DLT rate of 80% seen in WISTERIA arm B (with weekly cisplatin 40 mg/m^2^). The RP2D in that study was 100 mg bd (bd on days one to three of weeks one, two, four, five, seven, and eight), which was higher than the likely tolerated for Group B of WISTERIA, had the trial continued (TITE-CRM recommended reduction from 100 mg bd). However, it is difficult to directly compare the two studies due to the different study populations and the different radiotherapy and cisplatin doses.

There have been few studies combining AZD1775 with concurrent chemoradiation. A phase I study of AZD1775 in combination with definitive chemoradiotherapy was previously conducted in patients with cervical cancers (NCT01958658) but the study was put on hold in 2018 and the outcome of this study has not been reported. A similar study was conducted with AZD1775 in combination with chemoradiotherapy in patients with cervical, upper vaginal and uterine cancers (NCT03345784) but was closed early due to clinically significant toxicity and slow accrual so failed to determine the RP2D of AZD1775. In patients with locally advanced pancreatic cancer, a dose escalation study determined the RP2D of AZD1775 to be 150 mg/day (od on days one, two, eight, and nine every 21 days) with four cycles of gemcitabine (1,000 mg/m^2^ days one and eight in 21-day cycle) plus radiation (administered concurrently for cycles two and three) [33]. There were eight patients (24% out of 34 enrolled patients) who experienced a DLT, including neutropenic sepsis/thrombocytopenia and abnormal liver function tests.

Unfortunately, HNSCC patients with ECS and/or positive margins requiring post-operative chemoradiotherapy have a very high locoregional recurrence rate with a three-year disease-free survival of only 45% [34]. Some of these patients even develop disease recurrence before starting adjuvant post-operative chemoradiotherapy, particularly in those with surgical complications leading to delay in wound recovery. This contributes to recruitment issues for this group of patients. If we were to design a similar study again, we could consider combining AZD1775 with postoperative radiotherapy for patients with resectable locally advanced HNSCC without high-risk features such as ECS and positive margin. By targeting this population, we would omit concurrent cisplatin chemotherapy and potentially avoid the excess toxicities seen in the combination of AZD1775 with chemoradiotherapy. This group of patients are still at high risk of recurrence (three-year disease-free survival of 71%) [34] and they are potentially easier to recruit since they are seen more frequently than those requiring concurrent chemoradiotherapy.

Despite promising anti-tumour activity reported in previously published clinical trials on AZD1775, the excess toxicities seen by the combination of AZD1775 with chemotherapy or concurrent chemoradiotherapy prevent the further development of AZD1775 in patients with resectable HNSCC who require post-operative chemoradiotherapy as shown in our study. AZD1775 appears to be better tolerated when combined with other non-chemotherapeutic novel agents, in particular immunotherapy. In a phase Ib study of AZD1775 and durvalumab conducted in patients with advanced solid tumours (NCT02617277), the treatment combination showed a good safety profile with fatigue (15%), nausea (9%), and diarrhoea (11%) the most common grade ≥3 AEs; only two DLTs were observed, namely nausea (*N* = 2) and diarrhoea (*N* = 1) [35]. The RP2D for AZD1775 was 150 mg bd (three days on, four days off; treatment days 15–17, 22–24) with durvalumab 1500 mg (D1 Q4W) and there was evidence of antitumor activity with a disease control rate of 36% [35]. Therefore, this combination could be tested as adjuvant maintenance treatment following the completion of post-operative chemoradiotherapy or radiotherapy for patients with resectable HNSCC or recurrent or metastatic HNSCC whose disease has progressed after previous immunotherapy.

Recruitment for the Group A window study was particularly challenging; primarily due to the challenges related to coordinating recruiting patients who required surgery when delays to surgery were deemed unacceptable and unethical. Moreover, in some NHS hospitals that opened WISTERIA, surgery and oncology treatments were often administered at different sites, representing a further coordination challenge. In addition to logistic issues, the other major drawbacks highlighted previously for window studies included clinician concern regarding potential safety issues, such as post-surgical wound complications, risk of disease progression from delayed definitive treatment and a probable lack of patient benefit in giving a short course of treatment [36]. We would recommend that these issues be explored, and proposed solutions identified before a new window of opportunity study is carried out to avoid similar challenges being repeated.

To maximise recruitment and, reduce suspension time between cohorts, whilst balancing safety and optimal patient allocation, we also implemented a practical recruitment strategy of allowing screening cohorts of up to five patients if the dose has previously been tested. However, as recruitment was so poor, the WISTERIA trial did not have the chance to make use of this flexible strategy. Though we recruited three patients in Cohort 2 for Group B, only two were eventually evaluable. In a typical standard dose-escalation design with three or six patients, we would have to replace any non-evaluable patient before any decision can be made. However, the TITE-CRM design can make inferences with flexible cohort sizes, which further highlights its advantages, particularly in settings with patients in advanced disease settings where non-evaluability is not a rare occurrence. Sensitivity analyses with treatment-adjusted TITE-CRM models allowed the proportion of treatment received by Group B participants to be accounted for as well as the duration of the DLT monitoring period completed. Findings indicated that the proportion of treatment received did not influence the outcome of the TITE-CRM model. With continual reassessment and updating of posterior probabilities of each patient’s DLT information, the precision of DLT estimates would be improved. Despite early closure, we have demonstrated that TITE-CRM is not only a feasible design that could be utilised effectively in a resource-constrained setting, but it also offers distinct benefits in terms of flexibility, accrual, and statistical inference. These lessons learnt could help to shape the design of future clinical studies in AZD1775 or other DDR agents.

Although WISTERIA did not complete the primary objectives due to slow recruitment and toxicities seen in combination with chemoradiotherapy, the modified TITE-CRM trial design used to determine the MTD in a complex patient population with flexible cohort sizes was the first of such conducted at a UK academic institution. TITE-CRM provides greater accuracy in its MTD determination compared to rule-based designs, whilst reducing trial duration. This dose-escalation strategy is suited to settings where the DLT observational period is long compared to the expected patient recruitment period, to allow for a reduction in accrual suspension. Implementing an early stopping criterion that ensured favourable statistical properties and the incorporation of clinicians’ perspectives on when to stop early when excessive DLTs were observed at the lower doses using the dose transition pathways tool to map out dose decisions in advance, further strengthened the utility of the design in practice [37].

### Supplementary information

Supplementary Appendix

## Data Availability

Participant data and the associated supporting documentation will be available within 6 months after the publication of this manuscript. Details of our data request process is available on the CRCTU website. Only scientifically sound proposals from appropriately qualified research groups will be considered for data sharing. The decision to release data will be made by the CRCTU Director’s Committee, who will consider the scientific validity of the request, the qualifications and resources of the research group, the views of the Chief Investigator and the trial steering committee, consent arrangements, the practicality of anonymising the requested data and contractual obligations. A data sharing agreement will cover the terms and conditions of the release of trial data and will include publication requirements, authorship and acknowledgements and obligations for the responsible use of data. An anonymised encrypted dataset will be transferred directly using a secure method and in accordance with the University of Birmingham’s IT guidance on encryption of data sets.

## References

[1] Cancer Research UK. Head and neck cancers statistics [Available from: https://www.cancerresearchuk.org/health-professional/cancer-statistics/statistics-by-cancer-type/head-and-neck-cancers. Last Accessed: 23-Jan-2023.

[2] Cooper JS, Pajak TF, Forastiere AA, Jacobs J, Campbell BH, Saxman SB, et al. Postoperative concurrent radiotherapy and chemotherapy for high-risk squamous-cell carcinoma of the head and neck. N Engl J Med. 2004;350:1937–44. 10.1056/NEJMoa032646 15128893 10.1056/NEJMoa032646

[3] Kang H, Kiess A, Chung CH. Emerging biomarkers in head and neck cancer in the era of genomics. Nat Rev Clin Oncol. 2015;12:11–26. 10.1038/nrclinonc.2014.192 25403939 10.1038/nrclinonc.2014.192

[4] Dillon MT, Good JS, Harrington KJ. Selective targeting of the G2/M cell cycle checkpoint to improve the therapeutic index of radiotherapy. Clin Oncol (R Coll Radiol). 2014;26:257–65. 10.1016/j.clon.2014.01.009 24581946 10.1016/j.clon.2014.01.009

[5] Guertin AD, Li J, Liu Y, Hurd MS, Schuller AG, Long B, et al. Preclinical evaluation of the WEE1 inhibitor MK-1775 as single-agent anticancer therapy. Mol Cancer Ther. 2013;12:1442–52. 10.1158/1535-7163.Mct-13-0025 23699655 10.1158/1535-7163.Mct-13-0025

[6] Magnussen GI, Holm R, Emilsen E, Rosnes AK, Slipicevic A, Flørenes VA. High expression of Wee1 is associated with poor disease-free survival in malignant melanoma: potential for targeted therapy. PLoS One. 2012;7:e38254 10.1371/journal.pone.0038254 22719872 10.1371/journal.pone.0038254 PMC3373567

[7] Mir SE, De Witt Hamer PC, Krawczyk PM, Balaj L, Claes A, Niers JM, et al. In silico analysis of kinase expression identifies WEE1 as a gatekeeper against mitotic catastrophe in glioblastoma. Cancer Cell. 2010;18:244–57. 10.1016/j.ccr.2010.08.011 20832752 10.1016/j.ccr.2010.08.011 PMC3115571

[8] Moser R, Xu C, Kao M, Annis J, Lerma LA, Schaupp CM, et al. Functional kinomics identifies candidate therapeutic targets in head and neck cancer. Clin Cancer Res. 2014;20:4274–88. 10.1158/1078-0432.Ccr-13-2858 25125259 10.1158/1078-0432.Ccr-13-2858 PMC4135446

[9] Wu Z, Doondeea JB, Gholami AM, Janning MC, Lemeer S, Kramer K, et al. Quantitative chemical proteomics reveals new potential drug targets in head and neck cancer. Mol Cell Proteomics. 2011;10:M111.011635 10.1074/mcp.M111.011635 21955398 10.1074/mcp.M111.011635 PMC3237086

[10] Hirai H, Iwasawa Y, Okada M, Arai T, Nishibata T, Kobayashi M, et al. Small-molecule inhibition of Wee1 kinase by MK-1775 selectively sensitizes p53-deficient tumor cells to DNA-damaging agents. Mol Cancer Ther. 2009;8:2992–3000. 10.1158/1535-7163.Mct-09-0463 19887545 10.1158/1535-7163.Mct-09-0463

[11] Do K, Doroshow JH, Kummar S. Wee1 kinase as a target for cancer therapy. Cell Cycle. 2013;12:3159–64. 10.4161/cc.26062 24013427 10.4161/cc.26062 PMC3865011

[12] Bridges KA, Hirai H, Buser CA, Brooks C, Liu H, Buchholz TA, et al. MK-1775, a novel Wee1 kinase inhibitor, radiosensitizes p53-defective human tumor cells. Clin Cancer Res. 2011;17:5638–48. 10.1158/1078-0432.Ccr-11-0650 21799033 10.1158/1078-0432.Ccr-11-0650 PMC3167033

[13] Caretti V, Hiddingh L, Lagerweij T, Schellen P, Koken PW, Hulleman E, et al. WEE1 kinase inhibition enhances the radiation response of diffuse intrinsic pontine gliomas. Mol Cancer Ther. 2013;12:141–50. 10.1158/1535-7163.Mct-12-0735 23270927 10.1158/1535-7163.Mct-12-0735

[14] Sarcar B, Kahali S, Prabhu AH, Shumway SD, Xu Y, Demuth T, et al. Targeting radiation-induced G(2) checkpoint activation with the Wee-1 inhibitor MK-1775 in glioblastoma cell lines. Mol Cancer Ther. 2011;10:2405–14. 10.1158/1535-7163.Mct-11-0469 21992793 10.1158/1535-7163.Mct-11-0469 PMC5753756

[15] Karnak D, Engelke CG, Parsels LA, Kausar T, Wei D, Robertson JR, et al. Combined inhibition of Wee1 and PARP1/2 for radiosensitization in pancreatic cancer. Clin Cancer Res. 2014;20:5085–96. 10.1158/1078-0432.Ccr-14-1038 25117293 10.1158/1078-0432.Ccr-14-1038 PMC4184968

[16] Osman AA, Monroe MM, Ortega Alves MV, Patel AA, Katsonis P, Fitzgerald AL, et al. Wee-1 kinase inhibition overcomes cisplatin resistance associated with high-risk TP53 mutations in head and neck cancer through mitotic arrest followed by senescence. Mol Cancer Ther. 2015;14:608–19. 10.1158/1535-7163.Mct-14-0735-t 25504633 10.1158/1535-7163.Mct-14-0735-t PMC4557970

[17] Van Linden AA, Baturin D, Ford JB, Fosmire SP, Gardner L, Korch C, et al. Inhibition of Wee1 sensitizes cancer cells to antimetabolite chemotherapeutics in vitro and in vivo, independent of p53 functionality. Mol Cancer Ther. 2013;12:2675–84. 10.1158/1535-7163.Mct-13-0424 24121103 10.1158/1535-7163.Mct-13-0424 PMC3897395

[18] Do K, Wilsker D, Ji J, Zlott J, Freshwater T, Kinders RJ, et al. Phase I study of single-agent AZD1775 (MK-1775), a Wee1 Kinase inhibitor, in patients with refractory solid tumors. J Clin Oncol. 2015;33:3409–15. 10.1200/jco.2014.60.4009 25964244 10.1200/jco.2014.60.4009 PMC4606059

[19] Harrington KJ, Billingham LJ, Brunner TB, Burnet NG, Chan CS, Hoskin P, et al. Guidelines for preclinical and early phase clinical assessment of novel radiosensitisers. Br J Cancer. 2011;105:628–39. 10.1038/bjc.2011.240 21772330 10.1038/bjc.2011.240 PMC3188925

[20] Sharma RA, Plummer R, Stock JK, Greenhalgh TA, Ataman O, Kelly S, et al. Clinical development of new drug-radiotherapy combinations. Nat Rev Clin Oncol. 2016;13:627–42. 10.1038/nrclinonc.2016.79 27245279 10.1038/nrclinonc.2016.79

[21] Cheung YK, Chappell R. Sequential designs for phase I clinical trials with late-onset toxicities. Biometrics. 2000;56:1177–82. 10.1111/j.0006-341x.2000.01177.x 11129476 10.1111/j.0006-341x.2000.01177.x

[22] Kong A, Good J, Kirkham A, Savage J, Mant R, Llewellyn L, et al. Phase I trial of WEE1 inhibition with chemotherapy and radiotherapy as adjuvant treatment, and a window of opportunity trial with cisplatin in patients with head and neck cancer: the WISTERIA trial protocol. BMJ Open. 2020;10:e033009 10.1136/bmjopen-2019-033009 32184305 10.1136/bmjopen-2019-033009 PMC7076237

[23] O’Quigley J, Pepe M, Fisher L. Continual reassessment method: a practical design for phase 1 clinical trials in cancer. Biometrics. 1990;46:33–48. 2350571 10.2307/2531628

[24] Cheung YK. Dose Finding by the Continual Reassessment Method: Chapman and Hall/CRC; 2011.

[25] Yap C, Craddock C, Quigley JO, Billingham L. P75: comparing the implementation of a modified continual reassessment method to a traditional 3+3 design in a Phase I acute myeloid leukaemia trial. Clinical Trials. 2013;10:S1–S88. 10.1177/1740774513497438 23983107 10.1177/1740774513497438

[26] Common Terminology Criteria for Adverse Events (CTCAE) v4.0 2010 [Available from: https://ctep.cancer.gov/protocoldevelopment/electronic_applications/ctc.htm#ctc_40. Last Accessed: 24-Aug-2022

[27] Aaronson NK, Ahmedzai S, Bergman B, Bullinger M, Cull A, Duez NJ. et al. The European Organization for Research and Treatment of Cancer QLQ-C30: A quality-of-life instrument for use in international clinical trials in oncology. J Natl Cancer Inst. 1993;85:365–76. 8433390 10.1093/jnci/85.5.365

[28] Bjordal K, de Graeff A, Fayers PM, Hammerlid E, van Pottelsberghe C, Curran D, et al. A 12 country field study of the EORTC QLQ-C30 (version 3.0) and the head and neck cancer specific module (EORTC QLQ-H&N35) in head and neck patients. EORTC Quality of Life Group. Eur J Cancer. 2000;36:1796–807. 10.1016/s0959-8049(00)00186-6 10974628 10.1016/s0959-8049(00)00186-6

[29] Chen AY, Frankowski R, Bishop-Leone J, Hebert T, Leyk S, Lewin J, et al. The development and validation of a dysphagia-specific quality-of-life questionnaire for patients with head and neck cancer: the M. D. Anderson dysphagia inventory. Arch Otolaryngol Head Neck Surg. 2001;127:870–6. 11448365

[30] Leijen S, van Geel RM, Pavlick AC, Tibes R, Rosen L, Razak AR, et al. Phase I study evaluating WEE1 inhibitor AZD1775 as monotherapy and in combination with gemcitabine, cisplatin, or carboplatin in patients with advanced solid tumors. J Clin Oncol. 2016;34:4371–80. 10.1200/jco.2016.67.5991 27601554 10.1200/jco.2016.67.5991 PMC7845944

[31] Méndez E, Rodriguez CP, Kao MC, Raju S, Diab A, Harbison RA, et al. A Phase I clinical trial of AZD1775 in combination with neoadjuvant weekly Docetaxel and Cisplatin before definitive therapy in head and neck squamous cell carcinoma. Clin Cancer Res. 2018;24:2740–8. 10.1158/1078-0432.Ccr-17-3796 29535125 10.1158/1078-0432.Ccr-17-3796 PMC6004241

[32] Chera BS, Sheth SH, Patel SA, Goldin D, Douglas KE, Green RL, et al. Phase 1 trial of adavosertib (AZD1775) in combination with concurrent radiation and cisplatin for intermediate-risk and high-risk head and neck squamous cell carcinoma. Cancer. 2021;127:4447–54. 10.1002/cncr.33789 34379792 10.1002/cncr.33789

[33] Cuneo KC, Morgan MA, Sahai V, Schipper MJ, Parsels LA, Parsels JD, et al. Dose escalation trial of the Wee1 Inhibitor Adavosertib (AZD1775) in combination with Gemcitabine and radiation for patients with locally advanced pancreatic cancer. J Clin Oncol. 2019;37:2643–50. 10.1200/jco.19.00730 31398082 10.1200/jco.19.00730 PMC7006846

[34] Patel MR, Falchook GS, Wang JS-Z, Imedio ER, Kumar S, Motlagh P, et al. Open-label, multicenter, phase I study to assess safety and tolerability of adavosertib plus durvalumab in patients with advanced solid tumors. J Clin Oncol. 2019;37:2562 10.1200/JCO.2019.37.15_suppl.2562 10.1200/JCO.2019.37.15_suppl.2562 PMC11762568 39560862

[35] Maxwell JH, Ferris RL, Gooding W, Cunningham D, Mehta V, Kim S, et al. Extracapsular spread in head and neck carcinoma: impact of site and human papillomavirus status. Cancer. 2013;119:3302–8. 10.1002/cncr.28169 23797868 10.1002/cncr.28169

[36] Schmitz S, Duhoux F, Machiels JP. Window of opportunity studies: Do they fulfil our expectations? Cancer Treat Rev. 2016;43:50–7. 10.1016/j.ctrv.2015.12.005 26827692 10.1016/j.ctrv.2015.12.005

[37] Yap C, Billingham LJ, Cheung YK, Craddock C, O’Quigley J. Dose transition pathways: the missing link between complex dose-finding designs and simple decision-making. Clinical Cancer Research. 2017;23:7440–7. 10.1158/1078-0432.Ccr-17-0582 28733440 10.1158/1078-0432.Ccr-17-0582

