# Supplementary material for: Results and lessons learnt from the WISTERIA phase I trial combining AZD1775 with cisplatin pre- or post-operatively in head and neck cancer
Source: BJC Rep. 2024 Jan 29;2:6. doi: 10.1038/s44276-023-00026-6 (PMC11357979; doi:10.1038/s44276-023-00026-6)
Supplement: Supplementary file 1 — Supplementary Appendix [file 44276_2023_26_MOESM1_ESM.docx]

# Supplementary Appendix A – Summary of the TITE-CRM model for MTD assessment

In Group A, the highest safe dose in combination with cisplatin was determined using a predefined target DLT probability of 25% for up to 42 days from the start of treatment, identified during testing 75 mg twice daily (bd), 100 mg bd, 125 mg bd and 150 mg bd AZD1775 for three days. In Group B, the maximum tolerated dose (MTD) in combination with cisplatin/radiotherapy using the target dose limiting toxicity (DLT) of 30% for up to 12 weeks from the start of treatment, identified testing 50 mg, 75 mg, 100 mg, and 125 mg bd for three days on days two, nine, 23 and 30 from radiotherapy start.

The MTDs of AZD1775 for both groups were expected to differ given the additional toxicities of radiotherapy in Group B. Conservative target DLT rates were selected to minimise the likelihood of compromising individual patient’s chances of receiving radical surgery and/or post-operative radiotherapy. The use of predefined DLTs and the subsequent AZD1775 dose management has been previously described.^1^

To maximise recruitment, reduce trial suspension time between cohorts, whilst balancing safety and optimal patient allocation, screening up to five patients per cohort was permitted if the dose had previously been tested. Recruited patients were allocated to the current recommended dose up to a maximum of five. Replacement of unevaluable patients was permitted.

The model was designed to allow updates after every two to three evaluable patients, with any subsequent eligible patients (not already receiving treatment) allocated to the latest recommended dose cohort. Subsequent cohorts were assigned a dose level using all the data observed until either the MTD is determined; the maximum sample size is reached; or the trial is stopped early due to unacceptable DLT levels at the lowest dose.

The TITE-CRM model was modified to allow for the early termination of either group by the addition of the following criteria:

- If there was a high probability that the posterior probability of DLT at the lowest dose was greater than the target DLT rate, indicating that the lowest dose was too toxic. If the model recommends early stopping due to this safety criteria, the TMG and Trial Safety Committee (TSC) would be alerted and the TSC, with support of any external evidence, would assess whether the trial should be stopped.
- The trial would be allowed to stop early, before the full recruitment of 21 patients if nine patients have already been allocated at the most current MTD, which would be the recommended dose level for the next cohort if the trial continues, in consultation with the TSC.

References

1. Kong A, Good J, Kirkham A, et al. Phase I trial of WEE1 inhibition with chemotherapy and radiotherapy as adjuvant treatment, and a window of opportunity trial with cisplatin in patients with head and neck cancer: the WISTERIA trial protocol. *BMJ Open* 2020; **10**(3): e033009.

# Supplementary Appendix B – Operating characteristics of the TITE-CRM design

The time-to-event continual reassessment method (TITE-CRM) design operating characteristics used during the WISTERIA trial are shown in Tables B.1 (Group A) and B.2 (Group B). Designs differ with respect to the prior dose limiting toxicity (DLT) probabilities used and specified target DLT probability (25% for Group A and 30% for Group B). Group A used an expected accrual rate of two recruits per month (28 days) with a DLT monitoring period of 42 days (with a minimum of 30 days); Group B used an expected accrual rate of three recruits per month (28 days) with a DLT monitoring period of 84 days (with a minimum of 56 days).

Information supplied in Tables B.1 and B.2 list results for each of six test scenarios based on 10,000 simulation trials of up to 21 recruits in cohorts of three. The design allows for stopping for excess toxicity if the toxicity rate at the lowest dose exceeded the target DLT rate with a probability of 88% for Group A and 91% for Group B, and stop for consensus if nine participants were allocated to the same dose level.

Simulations were performed using the R software and ‘dfcm’ and ‘dtpcrm’ packages. Both Groups A and B have four dose levels to be assessed for the maximum tolerated dose (MTD), with corresponding estimated prior DLT probabilities given. The prior variance was set at one for both Group A and Group B simulations. For each scenario, the true toxicity level under test is given with simulation results for P(select) denoting the probability that a given dose combination level is selected as the MTD and the mean number of participants that would be assigned to that dose (numbers have been rounded to the nearest integer).

## Group A

### Table B.1: Operating characteristics of the TITE-CRM design for Group A

| **Scenario** |  | **Stop for Excess Toxicity** | **Consensus Reached (*N*=9)** | **Dose Levels** | | | |
| --- | --- | --- | --- | --- | --- | --- | --- |
|  |  |  |  | **-1** | **0**  **(starting dose)** | **1** | **2** |
|  | *Prior DLT Probabilities* |  |  | *0.02* | *0.06* | *0.14* | ***0.25*** |
| GroupA_TD25_1 | True Toxicity |  |  | **0.25** | 0.35 | 0.45 | 0.55 |
|  | P(select) | 0.07 | 0.89 | **0.47** | 0.34 | 0.11 | 0.01 |
|  | Mean Number of Participants |  |  | 5.10 | 6.40 | 2.78 | 0.46 |
| GroupA_TD25_2 | True Toxicity |  |  | 0.10 | **0.25** | 0.35 | 0.45 |
|  | P(select) | 0.01 | 0.95 | 0.16 | **0.48** | 0.27 | 0.08 |
|  | Mean Number of Participants |  |  | 2.75 | 6.78 | 4.54 | 1.42 |
| GroupA_TD25_3 | True Toxicity |  |  | 0.05 | 0.10 | **0.25** | 0.40 |
|  | P(select) | 0.00 | 0.96 | 0.01 | 0.20 | **0.52** | 0.27 |
|  | Mean Number of Participants |  |  | 0.51 | 5.17 | 6.79 | 3.80 |
| GroupA_TD25_4 | True Toxicity |  |  | 0.01 | 0.05 | 0.10 | **0.25** |
|  | P(select) | 0.00 | 0.96 | 0.00 | 0.02 | 0.19 | **0.78** |
|  | Mean Number of Participants |  |  | 0.10 | 3.65 | 5.20 | 7.50 |
| GroupA_TD25_5 | True Toxicity |  |  | 0.10 | 0.15 | 0.20 | **0.25** |
|  | P(select) | 0.00 | 0.95 | 0.05 | 0.20 | 0.30 | **0.45** |
|  | Mean Number of Participants |  |  | 0.99 | 5.10 | 5.36 | 4.46 |
| GroupA_TD25_6 | True Toxicity |  |  | 0.50 | 0.60 | 0.70 | 0.80 |
|  | P(select) | 0.58 | 0.41 | 0.39 | 0.02 | 0.00 | 0.00 |
|  | Mean Number of Participants |  |  | 5.79 | 4.43 | 0.45 | 0.01 |

DLT, dose limiting toxicity; P(select), probability of selecting that dose as the correct dose.

In scenarios 1-5 tested for the Group A design, the model correctly selected the MTD with probabilities ranging from 47% to 78%. The probability of choosing a dose with a true probability of DLT of higher than 25% (e.g., 30%) was no higher than 27%. The probability of stopping the trial due to excess toxicity was between 0% to 0.07%, whereas the probability of reaching a consensus for scenarios 1-5 was between 89% to 96%.

Scenario 6 has each true toxicity set to be too toxic and tests whether the model would stop due to excess toxicity. Simulation results found that the design would stop 58% due to excess toxicity. The allocation consensus supports the stopping rule stipulated to stop the trial in consultation with the oversight committee if nine participants were allocated to the same dose level (indicating that is likely the MTD).

It is possible to calculate in advance all feasible dose combinations that would be recommended by the model-based design if we have full DLT follow-up information. Dose transition pathways (DTP) illustrate the model decisions based on the number of DLTs recorded after each cohort, which in turn drives the decision as to whether the next cohort should receive an escalated dose, a de-escalated dose, remain on the current cohort’s dose or stop the trial early (Fig. B.1).


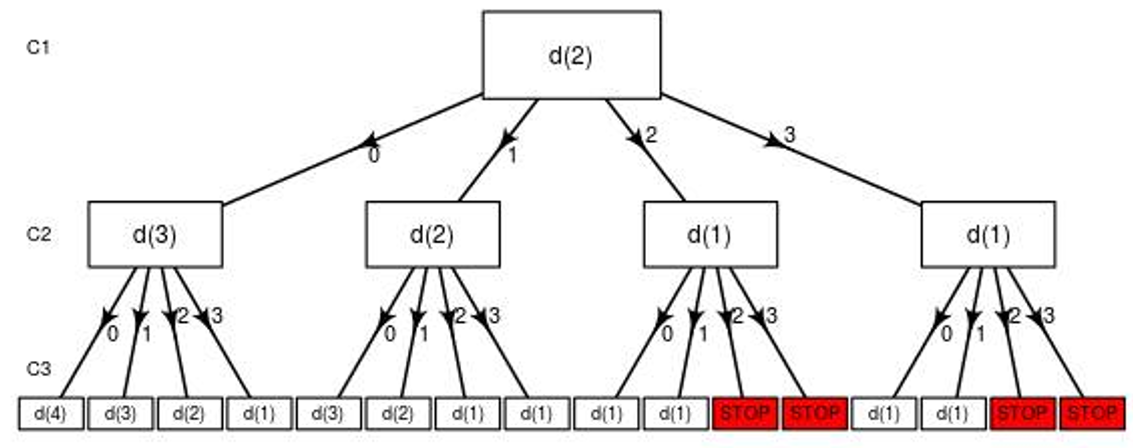


### Fig. B.1: Group A dose transition pathway

The DTP for Group A starting at dose level 2 (d(2)), has been calculated using the design parameters and uses cohorts of three participants (C1-C3). The arrows from the d(2) box for cohort C1 indicate all of the possible numbers of DLTs being observed (0 – 3). Based on these data, the trial design will recommend the next dose level to be allocated to the following new cohort. This is then repeated for cohorts C2 and C3. For example, if no DLTs were observed in cohort C1 the dose would be escalated up to dose level 3 for cohort C2; whereas if three DLTS were observed in cohort C1, the dose would be de-escalated to dose level 1 for cohort C2. The red boxes show when the model will recommend stopping the trial early when there is sufficient evidence that the lowest dose is too toxic.

## Group B

### Table B.2: Operating characteristics of the TITE-CRM design for Group B

| **Scenario** |  | **Stop for Excess Toxicity** | **Consensus Reached (*N*=9)** | **Dose Levels** | | | |
| --- | --- | --- | --- | --- | --- | --- | --- |
|  |  |  |  | **-1** | **0**  **(starting dose)** | **1** | **2** |
|  | *Prior DLT Probabilities* |  |  | *0.12* | *0.20* | ***0.30*** | *0.40* |
| GroupB_TD25_1 | True Toxicity |  |  | **0.30** | 0.40 | 0.50 | 0.60 |
|  | P(select) | 0.12 | 0.82 | **0.53** | 0.24 | 0.09 | 0.01 |
|  | Mean Number of Participants |  |  | 6.18 | 5.32 | 2.71 | 0.53 |
| GroupB_TD25_2 | True Toxicity |  |  | 0.20 | **0.30** | 0.40 | 0.50 |
|  | P(select) | 0.04 | 0.88 | 0.28 | **0.35** | 0.26 | 0.08 |
|  | Mean Number of Participants |  |  | 4.46 | 5.90 | 4.37 | 1.53 |
| GroupB_TD25_3 | True Toxicity |  |  | 0.05 | 0.20 | **0.30** | 0.40 |
|  | P(select) | 0.01 | 0.90 | 0.02 | 0.27 | **0.42** | 0.29 |
|  | Mean Number of Participants |  |  | 2.16 | 5.34 | 5.98 | 3.66 |
| GroupB_TD25_4 | True Toxicity |  |  | 0.01 | 0.05 | 0.10 | **0.30** |
|  | P(select) | 0.00 | 0.96 | 0.00 | 0.01 | 0.13 | **0.86** |
|  | Mean Number of Participants |  |  | 0.39 | 3.23 | 4.73 | 8.21 |
| GroupB_TD25_5 | True Toxicity |  |  | 0.10 | 0.20 | **0.30** | 0.40 |
|  | P(select) | 0.01 | 0.90 | 0.06 | 0.24 | **0.40** | 0.29 |
|  | Mean Number of Participants |  |  | 2.32 | 5.25 | 5.77 | 3.60 |
| GroupB_TD25_6 | True Toxicity |  |  | 0.50 | 0.60 | 0.70 | 0.80 |
|  | P(select) | 0.51 | 0.47 | 0.46 | 0.02 | 0.00 | 0.00 |
|  | Mean Number of Participants |  |  | 6.19 | 3.55 | 0.79 | 0.04 |

DLT, dose limiting toxicity; P(select), probability of selecting that dose as the correct dose.

In scenarios 1-5 tested with the Group B design, the model correctly selected the MTD with probabilities ranging from 35% to 86%. The probability of choosing a dose with a true probability of DLT of higher than 30% (e.g., 40%) was no higher than 24%. The probability of stopping the trial due to excess toxicity was between 0% to 0.12%, whereas the probability of reaching a consensus for scenarios 1-5 was between 82% to 96%. The allocation consensus would be flagged to the oversight committee to allow for the possibility of stopping the trial early. Scenario 6 has each true toxicity set to be too toxic and tests whether the model would stop due to excess toxicity. Simulation results found that the design would stop 51% due to excess toxicity.

The DTP has also been constructed for Group B using the Group B Trial design parameters (Fig. B.2).


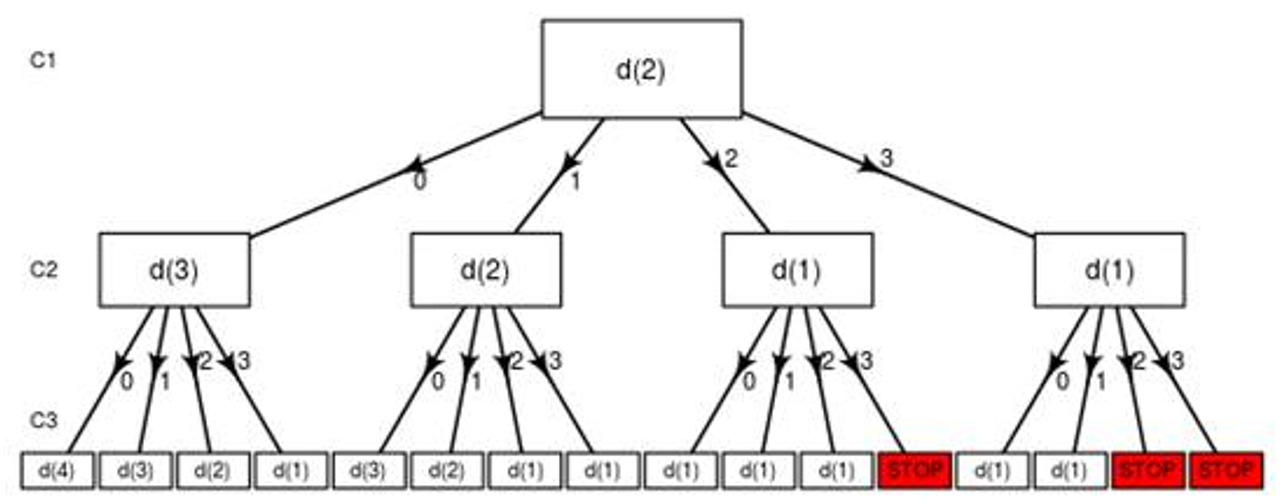


### Fig. B.2: Group B dose transition pathway

The DTP for Group B starting at dose level 2 (d(2)), has been calculated using the design parameters and uses cohorts of three participants (C1-C3). The arrows from the d(2) box for cohort C1 indicate all of the possible numbers of DLTs being observed (0 – 3). Based on these data, the trial design will recommend the next dose level to be allocated to the following new cohort. This is then repeated for cohorts C2 and C3. For example, if no DLTs were observed in cohort C1 the dose would be escalated up to dose level 3 for cohort C2; whereas if three DLTs were observed in cohort C1, the dose would be de-escalated to dose level 1 for cohort C2. The red boxes show when the model will recommend stopping the trial early when there is sufficient evidence that the lowest dose is too toxic.

# Supplementary Appendix C – Treatment-adjusted TITE-CRM sensitivity analysis

The TITE-CRM algorithm considers the occurrence of a DLT as yes/no (1 or 0) and includes a weighting for the proportion of DLT monitoring time (either as 1 if a DLT occurs, or as a proportion of time accrued), however, it does not account for the amount of treatment received. As part of a sensitivity analysis, we assessed whether weighting the algorithm to account for both the proportion of time and treatment received would influence its dose-decision making. The R package, dfcrm, used for the TITE-CRM calculations takes no account of the amount of treatment received but the options within this package can be changed (e.g., weight, split, etc), and taking advantage of this, we amended the weight option to account for the proportion of treatment received and DLT follow-up time for each patient using the following code:

Weight[i] <- (split[1]*(DosesTaken[i]/TotalDose) + split[2]*(FUpTime[i]/obswin)))

Here we split the weight option 50:50 to account for the dose received and DLT monitoring time (to keep the total weight to sum to 1). The drawback of this is that it assumes the treatment-to-toxicity distribution is uniform, and the dose received, and time accrued are equally important. Using a 40:60 ratio provides more weighting to the DLT monitoring period. Both 50:50 and 40:60 ratios were utilised in the sensitivity analyses.

**Application to the WISTERIA Group B data:**

A sensitivity analysis was carried out to assess any potential impact the reduction in the AZD1775 treatment dosage received by patients could have on the performance of the TITE-CRM model (in addition to the weights attributed based on the proportion of the full observation period (84 days) that a patient has been observed). The TITE-CRM was modified to take account of the proportion of the treatment received together with the DLT monitoring time each patient had and was run using equal weighting (50:50) for dose received and DLT monitoring follow-up time, and then again using a weighting of 40:60. This approach was not applicable to Group A data as all participants completed their scheduled treatments and DLT monitoring period assessment times.

# Supplementary Appendix D – Treatment and trial pathways of eligible patients


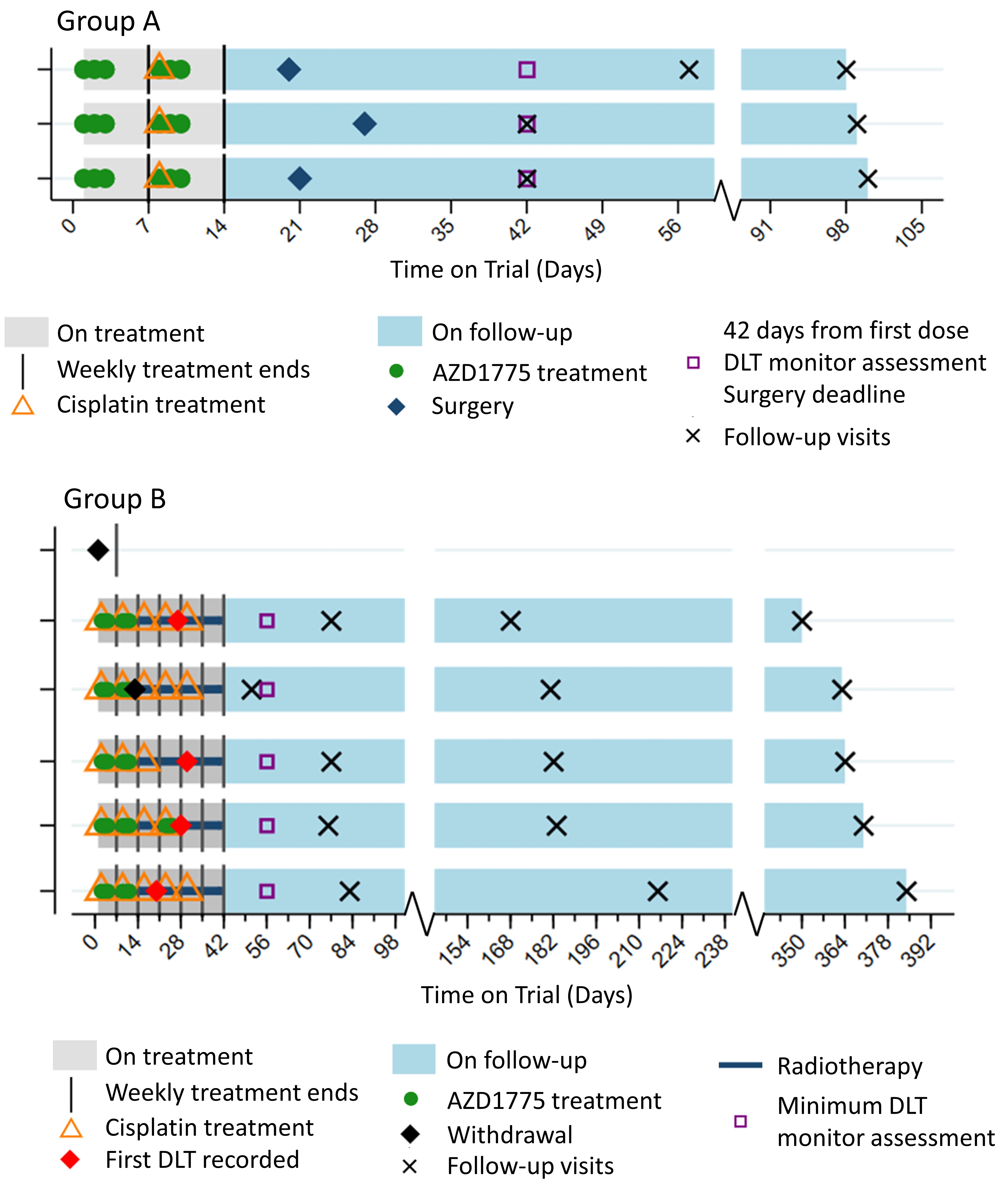


### Fig. D.1: Treatment and trial pathways o f eligible patients

Swimmer plots detailing the treatment and trial pathways of patients registered to the WISTERIA trial. See Figure 2 for the detailed treatment pathways.

All patients in Group A received 100 mg AZD1775 bd for three days during weeks one and two.

Four patients in Group B received 75 mg AZD1775 bd for three days during weeks one and two. One patient received 75 mg AZD1775 bd for three days during weeks one, two, and four. One patient withdrew from the trial prior to receiving any treatment.

# Supplementary Appendix E – Modified time-to-event continual reassessment model

Dose recommendation after Group A cohort 1 DLT assessment

Table E.1 provides an overview of the doses received by the patients recruited in Group A, occurrence of DLT and proportion of DLT follow-up time.

### Table E.1: Per patient treatment doses, occurrence of DLTs and proportion of DLT assessment period completed

| Days on Trial | Cohort | Dose Level | DLT | Proportion of DLT Assessment Period |
| --- | --- | --- | --- | --- |
| 100 | 1 | 0 | 0 | 1 (42/42) |
| 99 | 1 | 0 | 0 | 1 (42/42) |
| 98 | 1 | 0 | 0 | 1 (42/42) |

The number of DLTs experienced at the starting dose level 0 for Group A Cohort 1 patients, together with the estimated prior and posterior probabilities of observing a DLT are presented in Table E.2. The posterior probabilities combine the prior estimates of a DLT with the observed trial data.

The dose level with the closest posterior probability estimate to the target DLT rate of 0.25 (25%) is dose level 2 (posterior probability = 0.120, 90% credible interval 0-0.568) (Table E.2). However, in adherence to the modified TITE-CRM design, which specifies that no untried doses are skipped, the next recommended dose was dose level 1 for subsequent recruitment of Cohort 2 patients in Group A.

### Table E.2: Group A dose levels, prior and posterior probabilities of DLTS for each dose level with associated 90% credible intervals, based on the modified TITE-CRM dose-toxicity model

| Dose Level | AZD1755 Dose (mg) | Prior DLT Rate | Number of Evaluable Patients | Number of DLTs | Posterior DLT Rate (90% CI) |
| --- | --- | --- | --- | --- | --- |
| -1 | 75 | 0.02 | 0* | 0 | 0.003 (0, 0.203) |
| 0  (Starting dose) | 100 | 0.06 | 3 | 0 | 0.014 (0, 0.317) |
| 1 | 125 | 0.14 | 0* | 0 | 0.050 (0, 0.448) |
| 2 | 150 | 0.25 | 0* | 0 | 0.120 (0, 0.568) |

* Indicates untested doses

The prior and posterior probability estimates for each dose level following the DLT assessment for Group A Cohort 1 (*N*=3) are also presented in Fig. E.1.


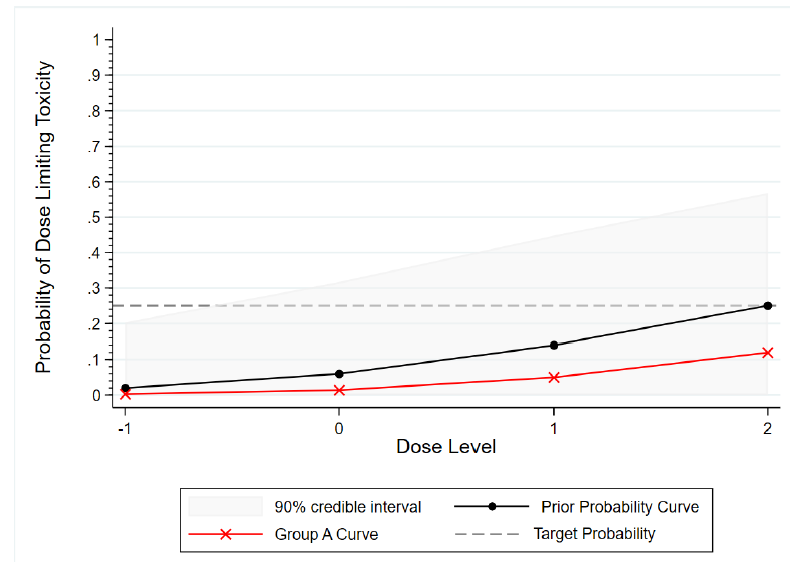


### Fig. E.1: Group A cohort 1 estimated DLT probabilities at each dose level

The dashed line illustrates the prior probability beliefs, and the red solid line illustrates the posterior probability estimates at each dose level. A reference line has been added to indicate the trial target DLT rate (0.25).

Dose recommendation after Group B (cohorts 1 and 2) DLT assessment

The TITE-CRM dose-toxicity model was updated following the completion of the DLT assessment period by all five evaluable Group B patients in Cohorts 1 and 2.

The number of DLTs experienced at the starting dose level 0 for Group B Cohorts 1 and 2 combined patients, together with the estimated prior and posterior probabilities of observing a DLT are presented in Table E.3 The posterior probabilities combine the prior estimates of a DLT with the observed trial data up to the end of the DLT assessment period. One participant was not evaluable and hence excluded from the TITE-CRM analysis as they did not receive any trial treatment.

### Table E.3: Per patient treatment doses, number of observed DLTs and proportion of DLT assessment period completed

| Days on Trial | Cohort | Dose Level | DLT | Proportion of DLT Assessment Period |
| --- | --- | --- | --- | --- |
| 384 | 1 | 0 | 1 | 1 |
| 364 | 1 | 0 | 1 | 1 |
| 363 | 1 | 0 | 0 | 0.607 (51/84) |
| 370 | 2 | 0 | 1 | 1 |
| 350 | 2 | 0 | 1 | 1 |
| 0 | 2 | 0 | - | - |

The dose level with the closest posterior probability estimate to the target DLT rate of 0.30 (30%) was dose level -1 (posterior probability = 0.525, 90% credible interval 0.179-0.786) (Table E.4). Therefore, the TITE-CRM model recommended de-escalating to dose level to -1 (the lowest possible dose) for the next cohort of Group B patients.

### Table E.4: Group B dose levels, prior and posterior probabilities of DLTS for each dose level with associated 90% credible intervals, based on the modified TITE-CRM dose-toxicity model

| Dose Level | AZD1755 Dose (mg) | Prior DLT Rate | Number of Evaluable Patients | Number of DLTs | Posterior DLT Rate (90% CI) |
| --- | --- | --- | --- | --- | --- |
| -1 | 50 | 0.12 | 0* | 0 | 0.525 (0.179, 0.786) |
| 0  (Starting dose) | 75 | 0.02 | 5 | 4 | 0.646 (0.310, 0.849) |
| 1 | 100 | 0.30 | 0* | 0 | 0.747 (0.458, 0.897) |
| 2 | 125 | 0.40 | 0* | 0 | 0.820 (0.588, 0.929) |

* Indicates untested doses

The prior and posterior probability estimates for each dose level following the DLT assessments for Group B Cohorts 1 (blue line) and combined Group B Cohorts 1 and 2 (red line), calculated using the TITE-CRM model are presented in Fig. E.2.


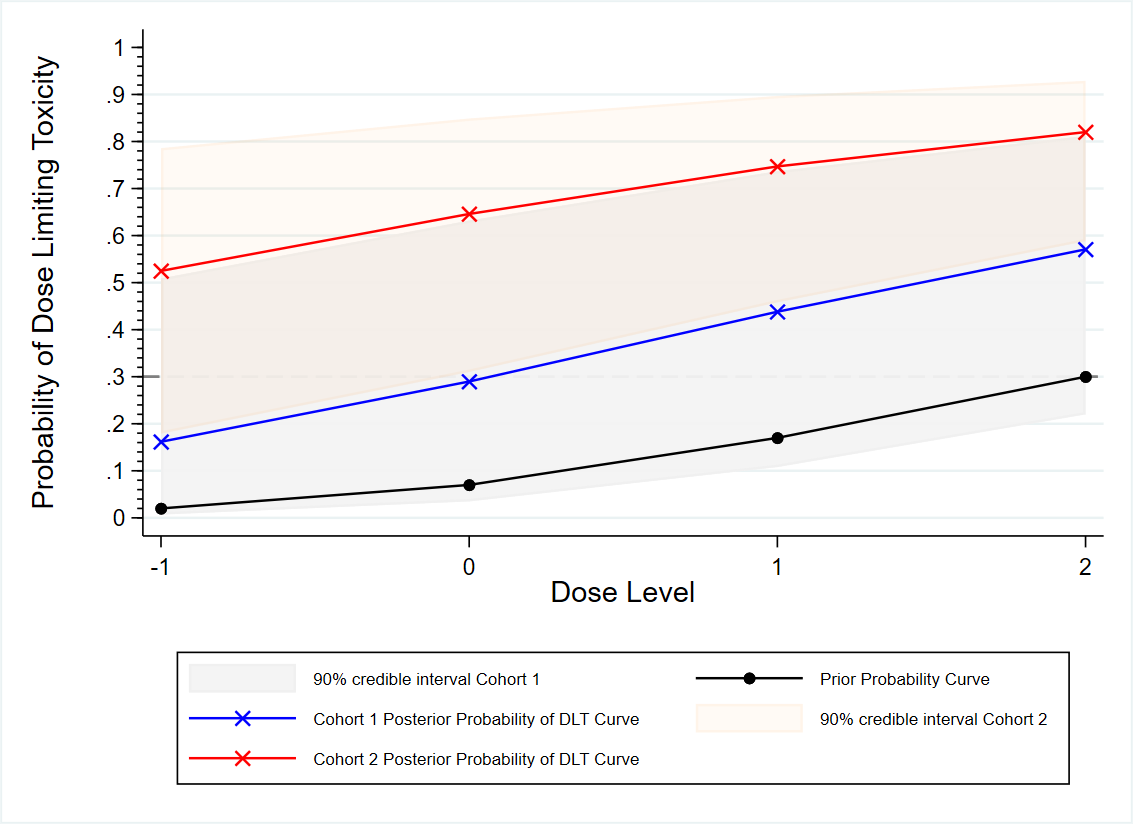


### Fig. E.2: Estimated DLT probabilities at each dose level, combined results for Group B Cohorts 1 and 2

The solid black line illustrates the prior probability beliefs, the red solid line illustrates the posterior probability estimates at each dose level (based on Group B cohort 1 and 2 data) with corresponding shaded 90% credible interval, and the dashed line denotes the reference line indicating the trial target DLT rate (0.30).

# Supplementary Appendix F – Pharmacokinetic data


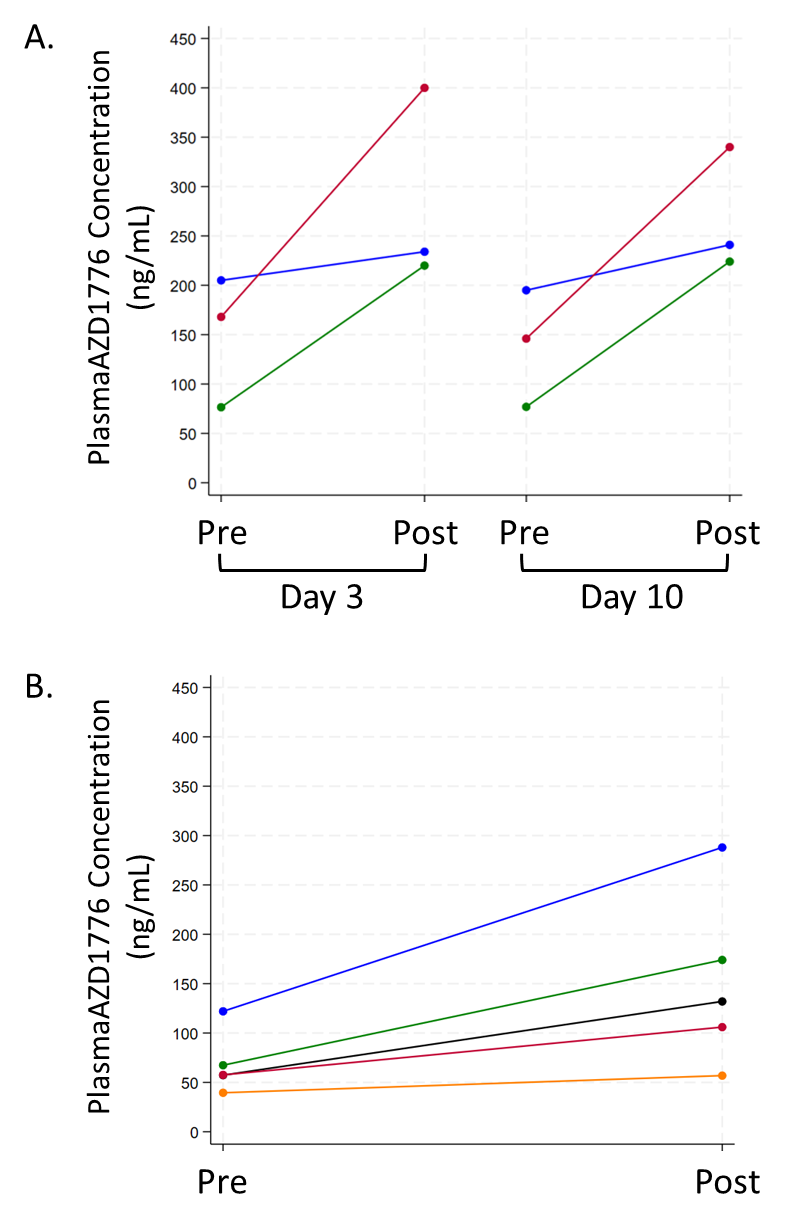


Fig. F.1: Plasma concentration of AZD1775 pre- and post-fifth dose of ASD1775

High performance liquid chromatography with tandem mass spectrometric detection was performed by Covance Laboratories Inc to determine AZD1775 concentration in the blood plasma of patients recruited into WISTERIA. Samples were collected pre- and post- the fifth dose of AZD1775 for all patients i.e., days three and ten for patients in Group A (A), and week one, day four for patients in Group B (B).

Individual patients are represented by different colours.

# Supplementary Appendix G – Quality of Life


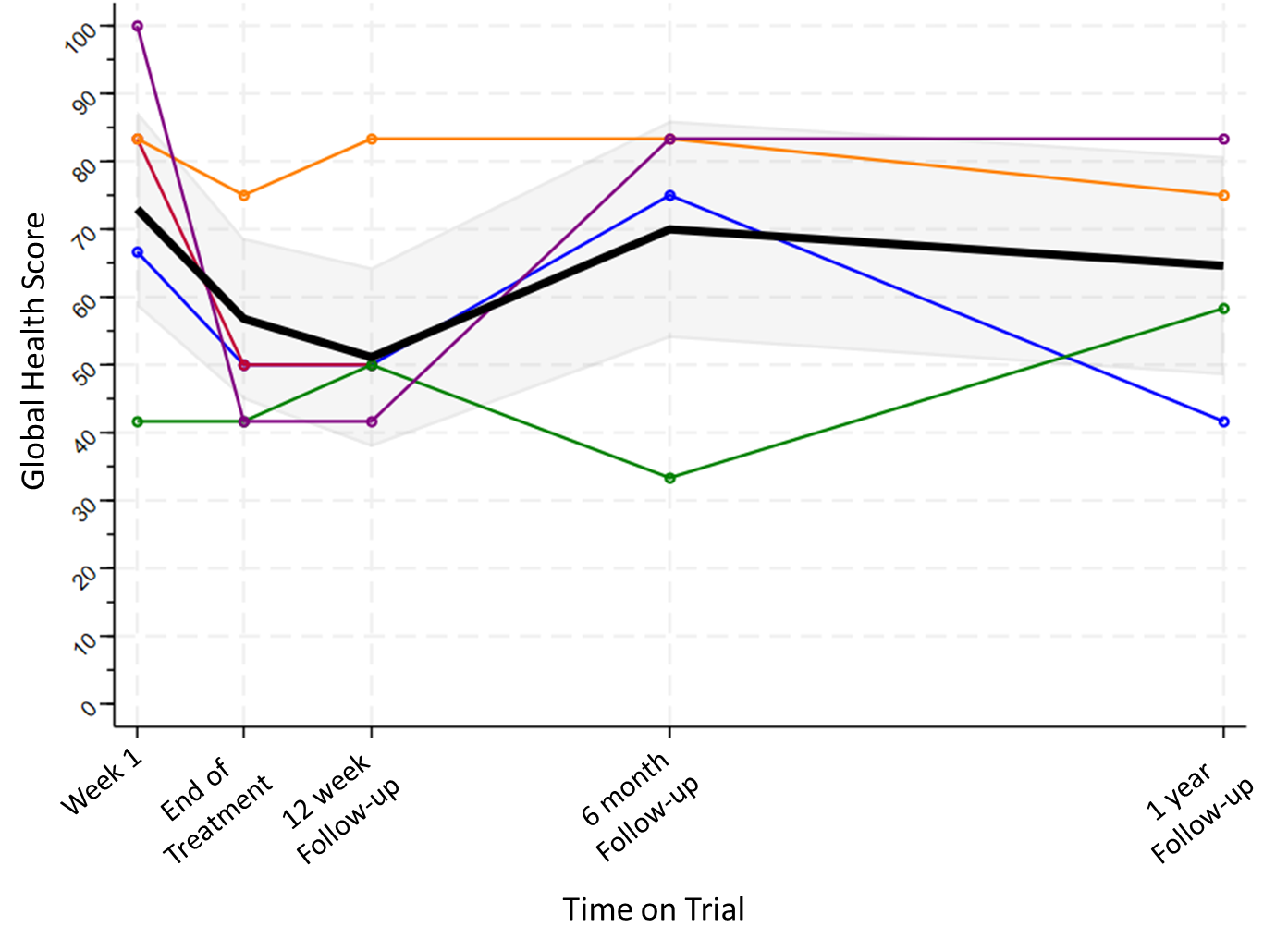


Fig. G.1: EORTC QLQ C30 global health scores

Quality of life (QoL) questionnaires were completed by patients recruited into Group B of the WISTERIA trial. Questionnaire data were completed independently by patients prior to commencement of radiotherapy, at the end of treatment assessment, and at the 12-week, six- and 12-month follow-up visits. Question responses were transformed into scores according to the instructions in the relevant questionnaire scoring systems.

Individual patients are represented by different colours. The mean trend line is shown as a solid black line with 95% uncertainty boundaries (grey shading).
